# Ultra-wide-angle multispectral narrow-band absorber for infrared spectral reconstruction

**DOI:** 10.1016/j.isci.2024.109700

**Published:** 2024-04-08

**Authors:** Yan Zheng, Liu Zhang, Ying Song, Jia-Kun Zhang, Yong-Nan Lu

**Affiliations:** 1 College of Instrumentation and Electrical Engineering, Jilin University, Changchun, Jilin 130012, China; 2 National Engineering Research Center of Geophysics Exploration Instruments, Jilin University, Changchun, Jilin 130061, China; 3 Institute of Electronics and Computer, Jilin Jianzhu University, Changchun, Jilin 130024, China

**Keywords:** Natural sciences, Physics, Optics

## Abstract

This paper presents the design of an ultra-wide-angle multispectral narrow-band absorber for reconstructing infrared spectra. The absorber offers several advantages, including polarization sensitivity, robustness against structural wear, wide azimuthal angle coverage, high narrow-band absorption, and adjustable working wavelength. To accomplish infrared spectrum reconstruction, an absorber is employed as a spectral sampling channel, eliminating the influence of slits or complex optical splitting elements in spectral imaging technology. Additionally, we propose using a truncation regularization algorithm based on the design matrix singular value ratio, namely IReg, which can enable high-precision spectral reconstruction under largely disturbed environments. The results demonstrate that, even when the number of absorption spectrum curve is reduced to a range of 1/2 to 1/3, high-precision spectral reconstruction is achievable for both flat and high-energy steep mid- and long-infrared spectral targets, while effectively accomplishing data dimension reduction.

## Highlights

• An absorber with anti-structural wear and wide azimuth coverage is designed
• Absorber used as spectral sampling channel for infrared spectral reconstruction
• IReg improves spectral reconstruction accuracy in largely disturbed environments
• High-precision reconstruction achieved with reduced data dimensionality

## Graphical abstract

**Figure:**
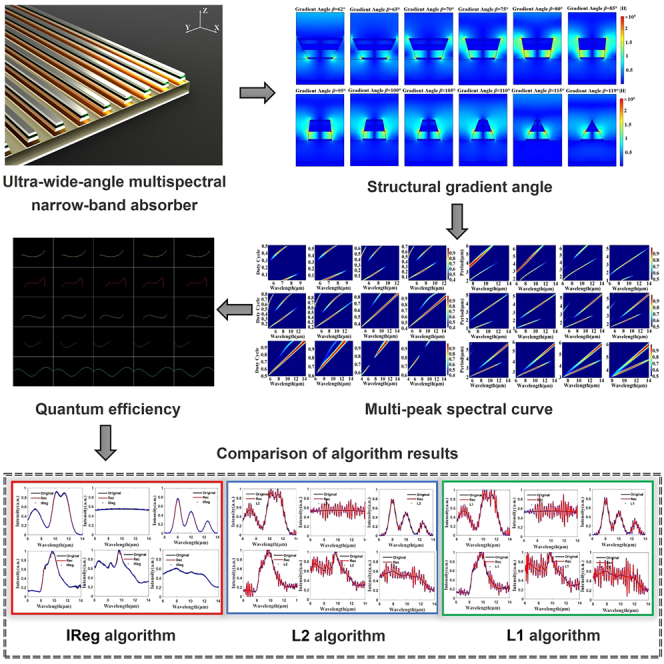

## Introduction

Metamaterial absorption filters based on micro-nano structures have the remarkable ability to efficiently capture and absorb incident electromagnetic waves at subwavelength scales.^1^^,^^2^ These absorbers are potentially applicable across various frequency regimes, including ultraviolet,^3^^,^^4^^,^^5^ visible,^4^^,^^5^^,^^6^^,^^7^ near-infrared,^8^^,^^9^^,^^10^^,^^11^ mid-long infrared,^8^^,^^12^^,^^13^ terahertz,^13^^,^^14^^,^^15^^,^^16^ and microwave^13^^,^^17^^,^^18^ frequencies. Enhancing the absorption efficiency of the infrared detector absorption layer is essential in improving detector performance. This improvement extends its utility to diverse areas, including optical absorption and sensing,^9^^,^^10^^,^^11^^,^^19^^,^^20^ solar wave absorption,^5^^,^^7^^,^^16^ microwave detection,^21^ visible spectrum detection,^22^ infrared spectrum detection,^23^^,^^24^ infrared stealth,^13^^,^^25^^,^^26^ and other fields.

Currently, the miniaturization of conventional spectrometers poses significant challenges due to considerations of size, cost, signal-to-noise ratios (SNRs), and spectral resolution. In longwave infrared spectroscopy, commonly used spectrometers include grating spectrometers^27^^,^^28^ and Fourier transform infrared spectrometers.^29^^,^^30^^,^^31^ However, the dependence of resolution on optical component size, optical path, or precision mechanical scanning equipment hinders miniaturization. To address the challenge of spectrometer miniaturization, a novel spectrometer based on a filter array and recovery algorithm has been recently developed,^32^^,^^33^^,^^34^^,^^35^^,^^36^^,^^37^^,^^38^^,^^39^^,^^40^^,^^41^^,^^42^^,^^43^^,^^44^^,^^45^^,^^46^^,^^47^^,^^48^^,^^49^^,^^50^^,^^51^^,^^52^^,^^53^^,^^54^ while most studies have focused on the visible^32^^,^^33^^,^^34^^,^^35^^,^^36^^,^^37^^,^^38^^,^^39^^,^^40^^,^^41^ to near-infrared^42^^,^^43^^,^^44^^,^^45^^,^^46^^,^^47^^,^^48^^,^^49^ spectrum, which is applied to objects like photonic chips,^42^^,^^49^ photonic crystal filters,^33^^,^^35^^,^^37^^,^^45^ superconducting single-photons,^46^ nanostructure arrays,^54^ nanowires,^38^^,^^47^ and quantum dot filters.^40^^,^^41^^,^^48^ However, the applicability of mid- to longwave infrared spectra to plasma metasurfaces,^50^ nanopore arrays,^51^ coaxial aperture arrays,^52^ and graphene plasma filters^53^ that are used to fabricate reconstruction spectrometer filters has been rarely explored. Notably, Lee et al.^51^ employed a nanopore array filter structure with an incidence between 55 % and 65 % over 2–10 μm to reconstruct spectral curves of two polymer materials using 50 sets of filter arrays. However, they only identified the positions of the characteristic peaks without performing a complete spectral curve reconstruction. Moreover, they did not perform spectral reconstruction under different SNR conditions. Craig et al.^52^ utilized a coaxial aperture array filter structure with a transmittance ranging between 50 % and 55 % over 6.2–14.2 μm and a degree of incidence varying within 10°. They only used a recursive least squares algorithm with 101 filter arrays to reconstruct the spectra of three samples and did not conduct any comparative analysis of the spectral reconstruction results at different SNRs. Dong et al.^53^ employed a graphene plasma filter structure along with a spectral reconstruction algorithm involving ridge regression and neural networks. They investigated the influence of graphene carrier mobility and SNR (40–80 dB) on spectral reconstruction. However, they did not consider spectral reconstruction for different target spectra or SNR cases. Therefore, by analyzing the aforementioned problems, we propose an ultra-wide-angle multispectral narrow-band absorber for infrared spectral reconstruction.

In addition, we compare and analyze the effect of target spectral curve reconstruction at different SNRs for a variety of simulation and ground targets in a large-disturbance environment (20–30 dB).

This study focuses on a metal-dielectric-metal (MDM) grating wear-insensitive ultra-wide-angle long-infrared multispectral narrow-band absorber, whose optical characteristics across the infrared spectrum (5–14 μm) do not vary with changes in structure, incidence, and orientation angles. The integration of a multispectral narrow-band absorption filter directly into the detector, while maintaining a high absorption efficiency, enables enhanced utilization of light energy, a high quantum efficiency, and a high spectral resolution. When an absorption filter is employed as a spectral sampling channel, the absorption filter spectral imaging technique allows spectrum recovery from channel measurements using a reconstruction method. This technique, combined with computational imaging spectroscopy, facilitates spectral reconstruction of detected targets without requiring slits or complex spectral structures; it offers advantages such as miniaturization, cost-effectiveness, and robustness to bias. The spectral curves employed in the spectral reconstruction are divided into narrow bands^43^^,^^51^^,^^52^^,^^53^^,^^54^^,^^55^ and broad bands.^40^^,^^41^^,^^56^^,^^57^^,^^58^ Narrow bands exhibit a singular value distribution in the matrix used to solve the linear equations; this characteristic reduces the condition number of the matrix and improves the accuracy of the linear equation solutions. To further examine the impact of spectral reconstruction, a detailed analysis of the reconstruction process was conducted.

Following the aforementioned analysis, this paper introduces a truncation regularization algorithm based on the design matrix singular value ratio, namely IReg algorithm. The accuracy of the spectral reconstruction, robustness of the linear equations, number of narrow-band spectral curves, and noise factors were taken into consideration. The robustness of the linear equations is correlated to the condition number of the design matrix,^59^ which significantly impacts the spectral reconstruction. Noise effects are also considered, as the spectrometer operation typically involves noise levels exceeding 30–80 dB.^41^^,^^57^^,^^60^ However, owing to detector noise, stray light, optical system aberration, and other error coupling, the error in the gray value is much greater than 30 dB.^61^ To address these challenges, a combination of Lasso regression (L1 regularization), ridge regression (L2 regularization), the IReg algorithm, and 22 sets of absorption spectral curves was used for spectral reconstruction corresponding to different simulated and ground objects under noise conditions ranging from 20 to 30 dB. The results demonstrate that, even when the absorption spectrum is reduced between 1/2 and 1/3, the spectra of both flat and high-energy steep mid- and long-infrared spectral targets can be reconstructed with a high precision, while effectively reducing data dimensions. This analysis reveals that combining the ultra-wide-angle long-infrared multispectral narrow-band absorber, based on a wear-insensitive MDM grating structure, with the IReg algorithm to correct and truncate the singular values of the design matrix enhances the robustness, accuracy, and precision of spectral reconstruction in the mid- and long-infrared bands.

## Results

### Structural design and simulation

**Figure 1:**
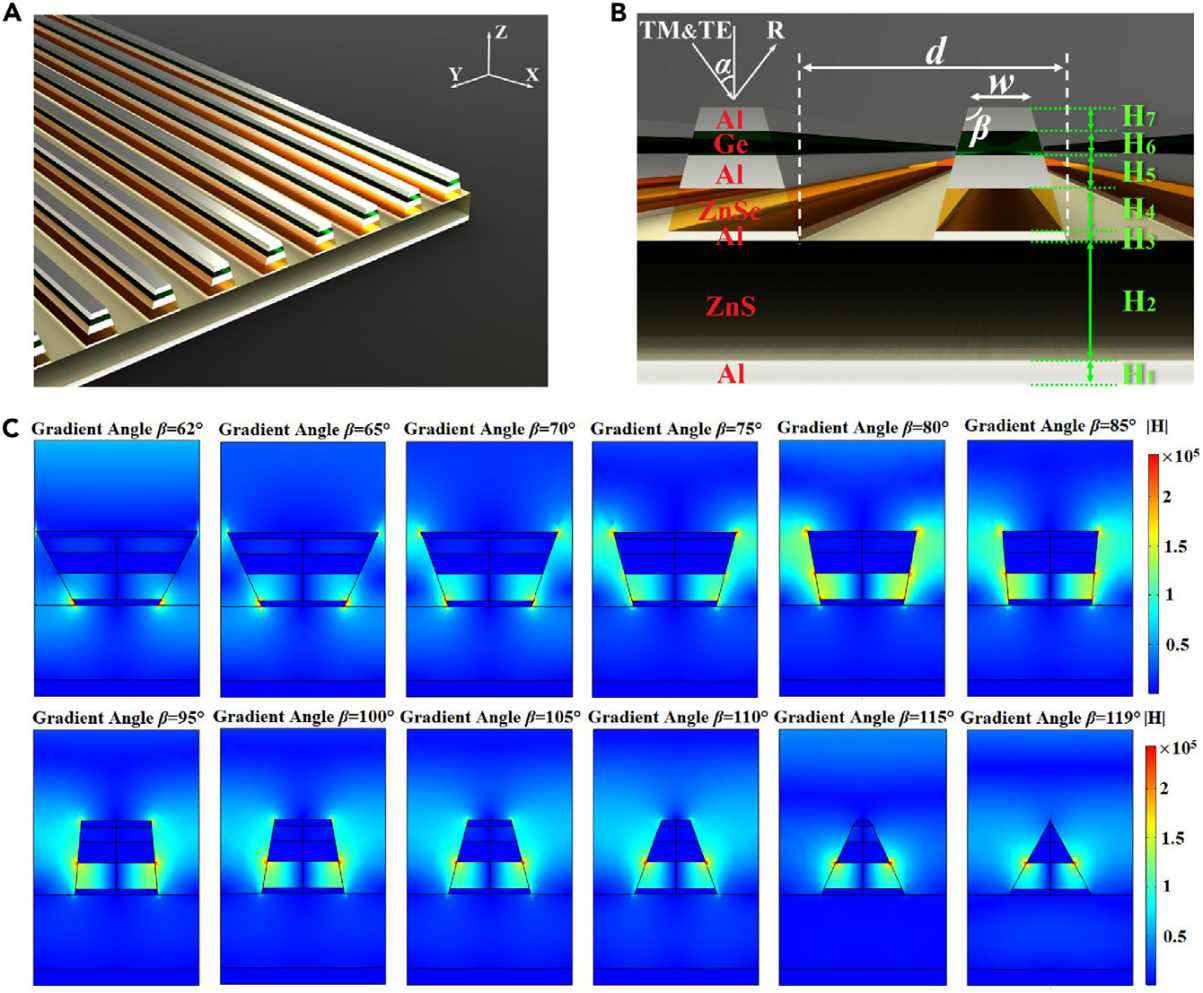
Structural design and parameter optimization (A) Schematic of an ultra-wide-angle long-infrared multispectral narrow-band absorber insensitive to the wear of the MDM grating structure based on the principle of surface plasmon resonance. (B) MDM grating structural parameters. (C) Variation of the electromagnetic field as β changes from 62° to 119°. (w=f·d, d=2μm, f=0.5, $H_{1}=200nm$, $H_{2}=900nm$, $H_{3}=70nm$, $H_{4}=322nm$, $H_{5}=248nm$, $H_{6}=182nm$, $H_{7}=76nm$).

At present, due to the actual processing and preparation of the grating structure, the angle between the grating structure and substrate changes. As a result, the phenomenon of grating structure angle β<90° or β>90° occurs, which affects the optical properties of optical devices, hinders engineering applications, and results in high costs. To solve the aforementioned problems, we designed an ultra-wide-angle multispectral narrow-band absorber, which was insensitive to structural angle changes and structural wear, for infrared spectral reconstruction as shown in Figure 1. This design incorporates the insensitivity to the MDM grating structure, and the finite element method was employed in this study to simulate various structural angles and achieve a composite narrow-band spectrum absorber with an improved long-band infrared spectrum detection performance. Among them, the surface plasmon resonance was excited using the grating compensation method, and the enhanced absorption effects of the MDM grating structure. The structure comprises aluminum (Al),^62^ germanium (Ge),^63^ zinc sulfide (ZnS),^64^ and zinc selenide (ZnSe) films,^64^ as depicted in Figures 1A and 1B. The width of the vertical cascade reactor is w=f·d, where *d* and f represent the periods of the structure and duty cycle, respectively. While maintaining a constant height parameter for the original structure, the structure angle *β* is adjusted to produce various gradient forms, which show the geometric model of the electromagnetic field mode distribution under different gradients in different directions; the structure angle *β* can range from 62° to 119°, as depicted in Figure 1C. By exploring the structure of the MDM grating and modulating the physical properties of the surface plasmon resonances, we can analyze the physical mechanism underlying the absorption effect. The subwavelength metal grating was used to achieve the polarization effect of the transverse magnetic (TM) wave transmission and the transverse electric (TE) wave shielding. Further, by considering the transmittance (T) of the MDM structure, the absorbance (A): A≡1-R-T can be readily derived from the reflectivity (R).

First, for the analysis of the grating structure angle *β*, we changed the shape of the grating structure by changing *β* from 62° to 119° from the minimum value to the maximum value. Second, we simulated and analyzed the optical properties of the absorber under extreme structural changes. The grating MDM structure of the absorber was simulated at a gradient of 62°–119° by selecting a partial gradient structure and further changing the duty cycle or period to obtain medium-long-wave infrared spectra, which resulted in multiple sets of absorption filter arrays. Finally, we combined the simulated absorption efficiency curves into 22 groups of absorption filter arrays and integrated them with the IReg algorithm to perform spectral reconstruction analysis on multiple target spectra. The analysis results demonstrated that infrared spectral curve reconstruction can still be applied under the ultra-wide-angle changes in the grating structure. Therefore, the tunable structure we designed can significantly reduce the difficulty of processing, improve the application rate of optical devices in engineering, and save costs; these are the objectives and advantages of our designed ultra-wide-angle narrower-band absorber for infrared spectral reconstruction.

**Figure 2:**
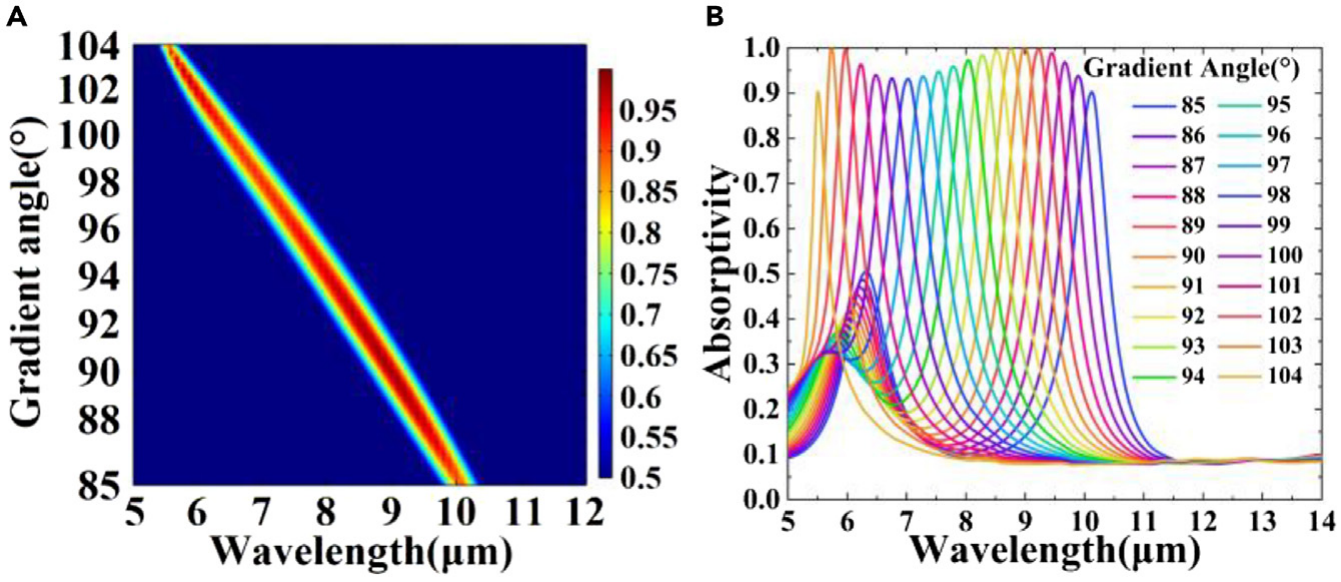
Absorption efficiency at gradient angles and under vertical incidence conditions (A and B) Absorption efficiency of over 90 % at gradient angles and under vertical incidence conditions.

**Figure 3:**
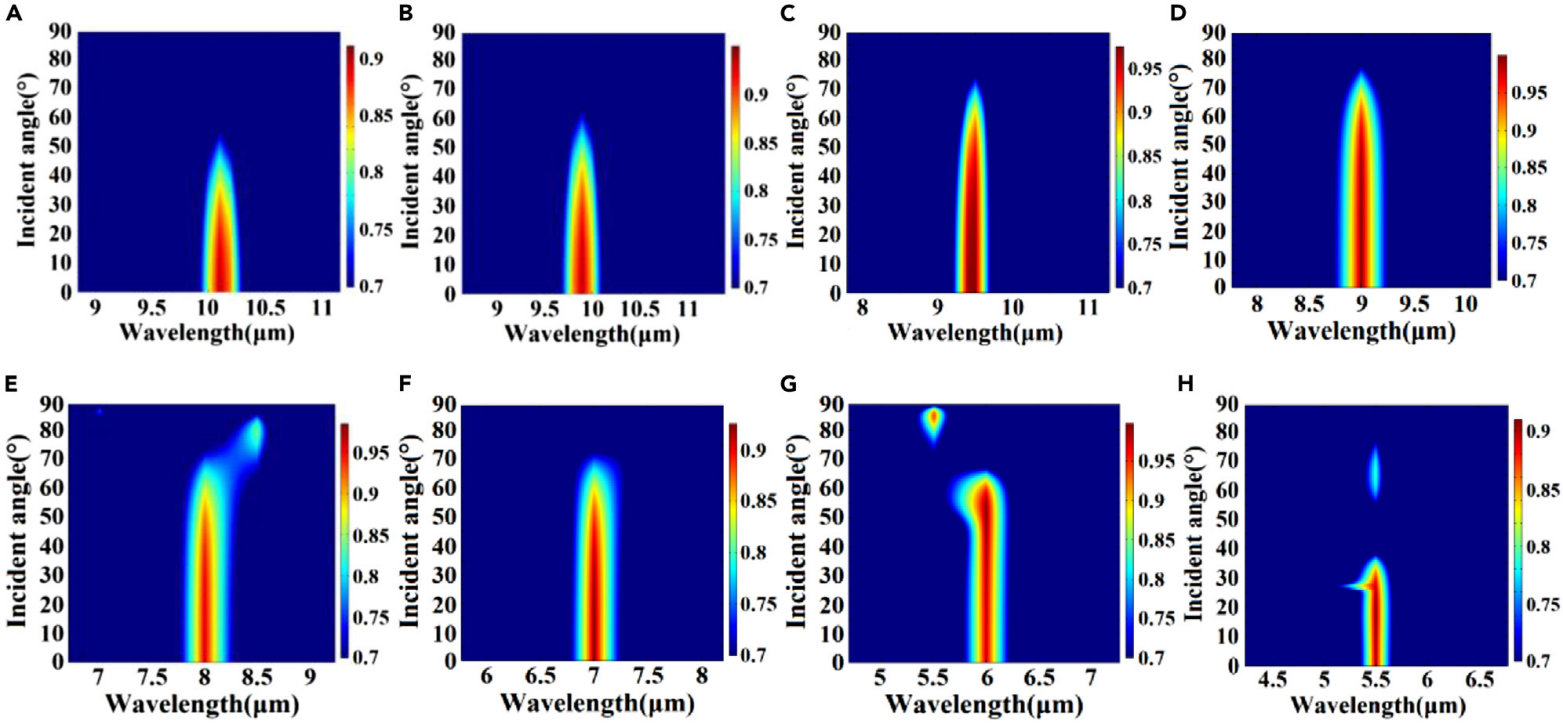
Spectral curves under incident angles of 0°–89° The gradient angles of (A) β=85°, (B) β=86°, (C) β=88°, (D) β=90°, (E) β=94°, (F) β=98°, (G) β=102°, and (H) β=104°.

**Figure 4:**
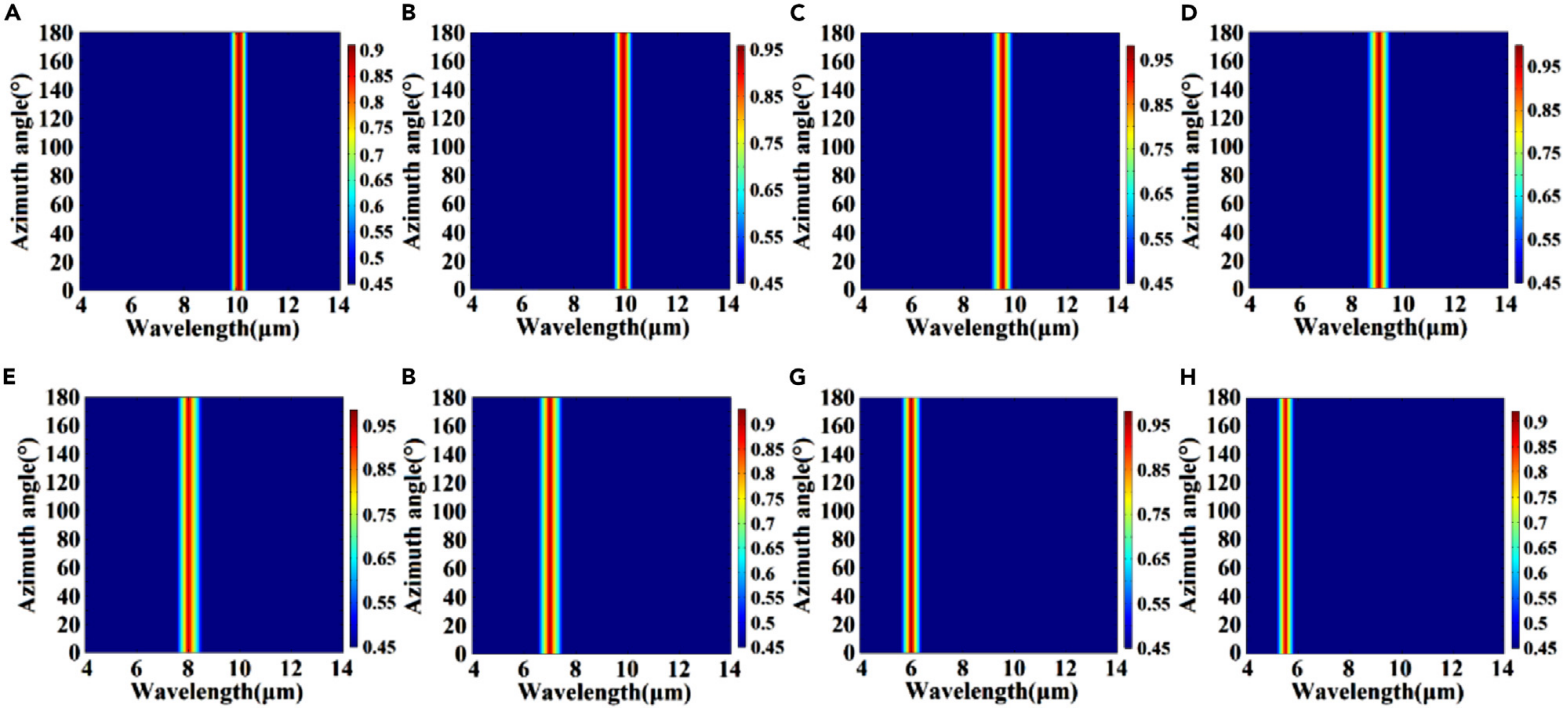
Spectral curves under azimuth angles of 0°–180° The gradient angles of (A) β=85°, (B) β=86°, (C) β=88°, (D) β=90°, (E) β=94°, (F) β=98°, (G) β=102°, and (H) β=104°.

**Figure 5:**
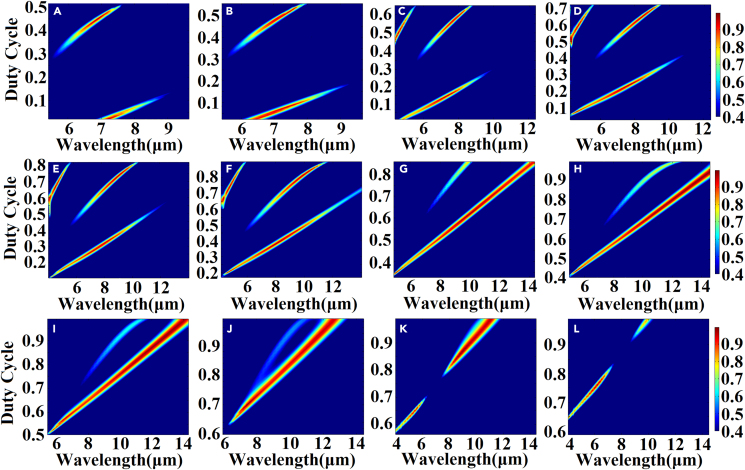
Relationship between absorption efficiency and duty cycle at structural gradient angles of *β* = 62°–119° The gradient angles of (A) β=62°, (B) β=65°, (C) β=70°, (D) β=75°, (E) β=80°, (F) β=85°, (G) β=95°, (H) β=100°, (I) β=105°, (J) β=110°, (K) β=115°, and (L) β=119°.

**Figure 6:**
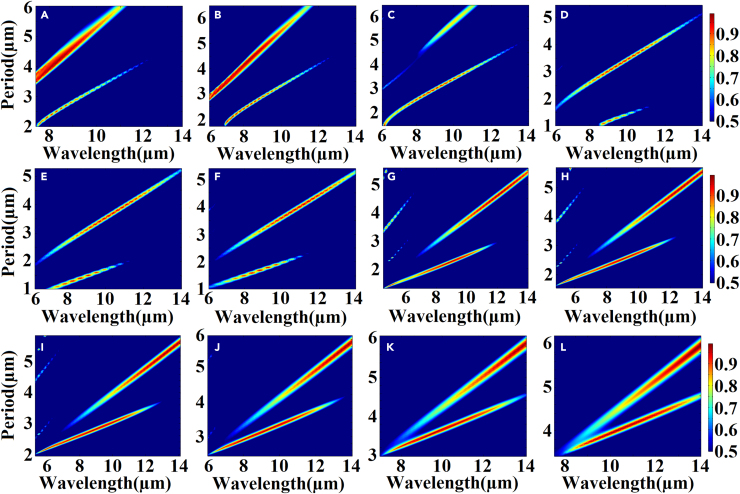
Relationship between absorption efficiency and period at structural gradient angles of *β* = 62°–119° The gradient angles of (A) β=62°, (B) β=65°, (C) β=70°, (D) β=75°, (E) β=80°, (F) β=85°, (G) β=95°, (H) β=100°, (I) β=105°, (J) β=110°, (K) β=115°, and (L) β=119°.

Hence, in this study, we examined the variations in the structural gradient angle *β*, the incidence angle α, and the azimuthal angle *θ.* Figures 2A and 2B illustrate the absorption efficiency of the 5.5–10.1 μm spectrum when the structural gradient angle *β* changes from 104° to 85°. Figures 3A–3H present the range of the structural gradient angle α from 0° to 89°, along with the structural gradient angles of β=85°, β=86°, β=88°, β=90°, β=94°, β=98°, β=102°, and β=104° at 10.1, 9.9, 9.5, 9, 8, 7, 6, and 5.5 μm as well as α at 10°, 34°, 58°, 66°, 54°, 44°, 62°, and 26°. And Figures 4A–4H show that, when we change the azimuth angle *θ* from 0° incidence to 180°, the absorption efficiencies exceed 90 % in all the cases, indicating that the absorber can operate effectively, surpassing the limitations of previous slit spectrometers. Moreover, this design significantly enhances the energy utilization of the spectrum detection system. A detailed data analysis is presented in Table 1. To obtain the spectra of different regions and wavelengths within the absorption filter array, overcoming the challenges associated with increasing the order of coating times in the traditional coating process and ensuring relative parallelism of multiple bands in the zone are necessary. In this design, the membrane thickness of each structure remains constant, while the structural gradient angle *β* changes from 119° to 62°. The optical characteristics of the structure were analyzed by changing the duty cycle or cycle of the structure to achieve controlled translation of the central wavelength of the absorption peak (Figures 5 and 6). Detailed data are presented in supplemental information.

**Table 1:** Relationship between incident angle and absorptivity for different gradients and wavelengths

Gradient angle (°)	Duty cycle	Period (μm)	Central wavelength (μm)	Vertical incidence absorption rates (%)	Incident angle (°)	Absorption rate (%)	Incident angle (°)	Absorption rate (%)
104	0.5	2	5.5	90.99	26	90.06	35	80.69
102	0.5	2	6	97.89	62	90.37	64	84.06
98	0.5	2	7	92.49	44	90.18	65	80.09
94	0.5	2	8	96.19	54	90.01	66	80.61
90	0.5	2	9	99.99	66	90.54	73	81.80
88	0.5	2	9.5	97.20	58	90.47	69	80.04
86	0.5	2	9.9	93.91	34	90.03	53	80.28
85	0.5	2	10.1	90.99	10	90.08	42	80.48

This approach improves the performance and quantum efficiency of the detector based on its absorption characteristics within the long-infrared spectral range. The absorption filter spectral imaging technique, which eliminates the need for slits or complex spectral structures, utilizes an absorption filter as a spectral sampling channel to recover spectrum from channel measurements using a reconstruction method. The absorber, in conjunction with computational imaging spectroscopy, facilitates spectral reconstruction of detected targets while offering several advantages, such as miniaturization, cost-effectiveness, and robustness against bias. Compared to the existing filter array and recovery algorithms employed in new miniaturization spectrometer research,^32^^,^^33^^,^^34^^,^^35^^,^^36^^,^^37^^,^^38^^,^^39^^,^^40^^,^^41^^,^^42^^,^^43^^,^^44^^,^^45^^,^^46^^,^^47^^,^^48^^,^^49^^,^^50^^,^^51^^,^^52^^,^^53^^,^^54^ the proposed MDM grating structure allows the use of ultra-wide-angle long-infrared multispectral narrow-band absorbers in reconstruction of long-infrared spectrometer spectra for various simulation and ground object targets, even under significant spectral disturbances. When errors occur during the processing and preparation of the structure due to the gradient of the grating structure, the spectral curve obtained by adjusting the duty cycle and period can accurately reconstruct the target spectrum.

**Figure 7:**
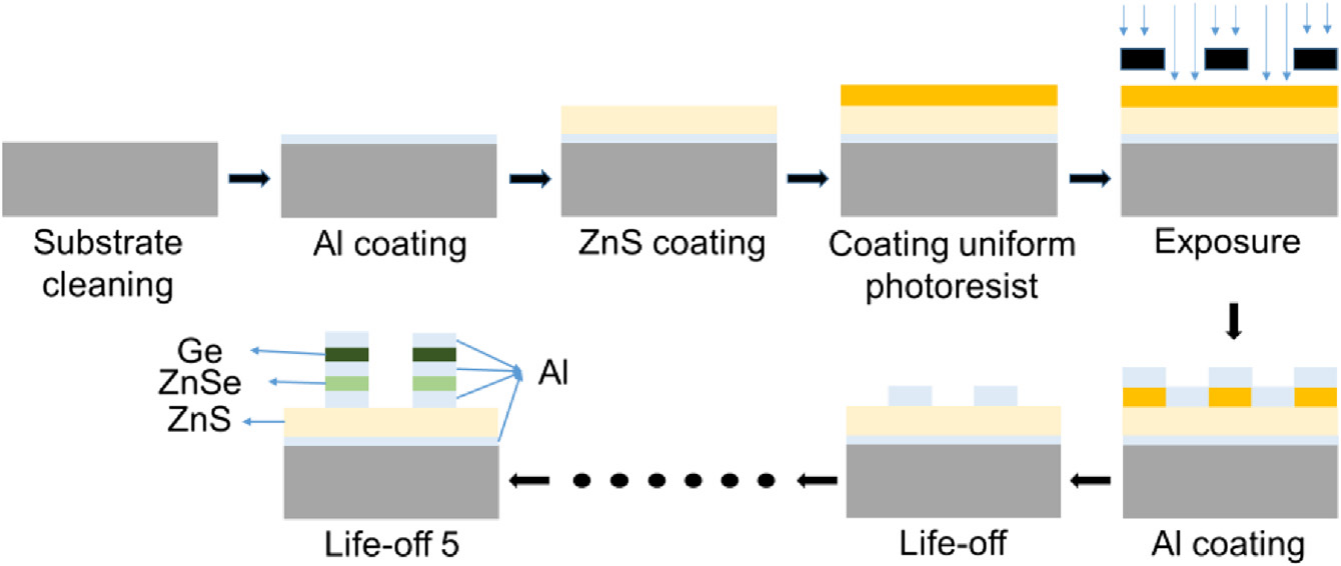
Schematic of the preparation process

Based on our prior preparation experience, the process used to create the grating MDM structures is as follows. Firstly, on the substrate material, Al and ZnS film were coated on the substrate surface by a magnetron sputtering coating process under vacuum in a dust-free environment. Subsequently, mask lithography was performed using a masking plate with different grating structure patterns, and the grating structure was photolithographed on the surface of the ZnS. Furthermore, Al, ZnSe, Al, Ge, and Al target materials were applied to the surface of the structure by the lithography and lift-off processes. The lift-off process involves soaking the coated substrate with photoresist in the degumming solution and peeling off the film outside the grating structure together with the photoresist to obtain a grating structure with an MDM film. The specific processing method must be refined after detailed process exploration. The schematic of the preparation process is shown in Figure 7.

Through the aforementioned preparation process, the processing accuracy can be optimized by iterative exploration of process parameters, and high-precision micro-nano structure optical devices can be obtained. The advantage of this process is that it can be performed in a large area to prepare micro-nano structures with low cost. If micro-nano structural optical devices with higher precision structural angle changes are desired, we can further use the focused-ion-beam (FIB) system for processing and preparation.

### Spectral reconstruction analysis

**Figure 8:**
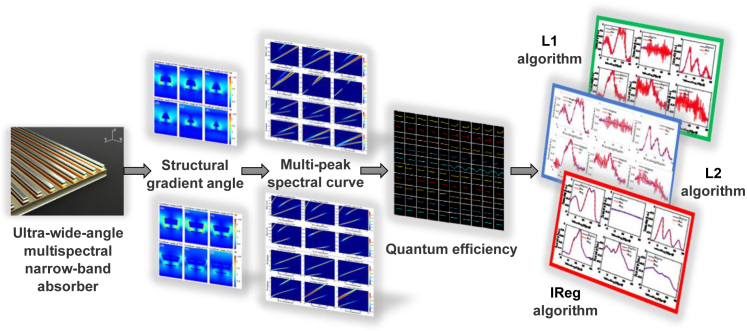
Schematic representation of the spectral reconstruction flow

An ultra-wide-angle long-infrared multispectral narrow-band absorber is proposed in this study based on the surface plasmon resonance principle, which combines the IReg algorithm to achieve a high-precision spectral reconstruction of various simulation targets and ground objects. In addition, this method is better than the L1 and L2 algorithms. This principle is shown in Figure 8. The infrared band spectrum reconstruction can be calculated from the target spectrum, the quantum efficiency curve with enhanced absorption efficiency, and the gray value of the detector. According to the earlier analysis, the structural gradient angle ranges from 62° to 119°. A total of 24 groups of absorption spectra were obtained. We select 22 groups to reconstruct different targets in the wavelength range of 5–14 μm.

A(λ) is the quantum efficiency curve of the absorption efficiency-enhanced type, X(λ) is the target spectrum, and the spectral reconstruction can be determined using Equation 1.

(Equation 1) $$\int_{\lambda_{1}}^{\lambda_{2}}A\left(\lambda \right)X\left(\lambda \right)d\lambda =B$$

Equation 1 is discretized to obtain Equation 2:

(Equation 2) A·X=B

Matrix ***A*** is referred to as the design matrix in this study.

However, the strong similarity of the absorption spectral curves of the 22 groups resulted in design matrix ***A*** being severely ill conditioned. When noise is generated, it causes a large error in the solution of the equations, thus affecting the accuracy of the spectral reconstruction. When the noise was greater than a certain critical value, the suppression effect of L1 and L2 regularization on noise was not significant. Thus, we propose an IReg algorithm that can achieve high-precision spectral reconstruction of a target in a large-disturbance environment.

Among them, L1 regularization has the same penalty on all parameters and can change part of the weights to zero, thus producing a small number of features and sparse models. The L1 regularization can solve the minimum problem using Equation 3.

(Equation 3) $$\min\left\{{\left\|AX-B\right\|}_{2}+\lambda {\left\|X\right\|}_{1}\right\}$$

The L2 regularization can solve the minimum problem using Equation 4, whose derivative is given by Equation 5.

(Equation 4) $$\min\left\{{\left\|AX-B\right\|}_{2}^{2}+\lambda {\left\|RX\right\|}_{2}^{2}\right\}$$

(Equation 5) $$X={\left(A^{T}A+\lambda R^{T}R\right)}^{-1}A^{T}B$$

In this algorithm, ***R*** is the regularization matrix and ***R*** is the unit matrix. Moreover, ***R*** corrects the singular value of the design matrix ***A***, and the regularization parameter determines the correction amplitude of the singular value, which can be obtained using the L-curve method. By decomposing design matrix ***A*** into singular values, the following equation can be obtained: $U=\left[u_{1},u_{2},u_{3},\cdots ,u_{n}\right]$, $S=\left[\sigma_{1},\sigma_{2},\sigma_{3},\cdots ,\sigma_{n}\right]$, and $V=\left[v_{1},v_{2},v_{3},\cdots ,v_{n}\right]$ can be obtained, where $u_{i}$ and $\nu_{i}$ are the left and right singular vectors, respectively, $\sigma_{i}$ is the singular value, and $f=\frac{\sigma_{i}^{2}}{\sigma_{i}^{2}+\lambda }$ is a filter function. Equation 5 can be reduced to

(Equation 6) $$X=\sum_{i=1}^{n}\frac{{\sigma_{i}}^{2}}{{\sigma_{i}}^{2}+\lambda }\cdot \frac{u_{i}^{T}v_{i}}{\sigma_{i}}b_{i}$$

In spectral reconstruction, the singular value size represents the information of the detected target spectrum. Moreover, the larger the singular value is, the more information it contains regarding the target spectrum. In L2 algorithm, the filter function corrects singular values to the same extent, but it is still not enough to achieve high-precision spectral reconstruction.

In IReg algorithm, we first design a regularization matrix ***G***, which aims to modify the larger singular values by a small margin and the smaller singular values by a large margin. Matrix ***G*** is represented by Equation 7.

(Equation 7) $$G=\left[\begin{array}{cccc} \log_{2}\left(1+\frac{\sigma_{1}}{\sigma_{1}}\right) & 0 & 0 & 0\\ 0 & \log_{2}\left(1+\frac{\sigma_{1}}{\sigma_{2}}\right) & 0 & 0\\ 0 & 0 & \ddots & 0\\ 0 & 0 & 0 & \log_{2}\left(1+\frac{\sigma_{1}}{\sigma_{n}}\right) \end{array}\right]$$

Substituting Equation 7 into Equation 6 yields a numerical analytical solution based on Equation 2 for the matrix ***G***, as shown in Equation 8.

(Equation 8) $$X=\sum_{i=1}^{n}\frac{{\sigma_{i}}^{2}}{\sigma_{i}^{2}+\lambda (\log_{2}{\left(1+\frac{\sigma_{1}}{\sigma_{i}}\right)}^{2}}\cdot \frac{{u_{i}}^{T}v_{i}}{\sigma_{i}}b_{i}$$

After the correction of matrix ***G***, the condition number of the design matrix is reduced, but the singular value is excessively corrected, and the spectral discrete points with small energy are excessively enlarged or reduced, thus affecting the accuracy of the spectral reconstruction. Second, we take the design matrix ***A*** singular value ratio as the truncated standard, determine the truncated threshold, and truncate the sequence of singular values to further reduce the number of conditions. To evaluate the degree of pathology during the design matrix ***A***, we used the conditions:

(Equation 9) $$Cond\left(A\right)=\frac{\sigma_{\max}}{\sigma_{\min}}$$

The number of conditions for the 22 sets of absorption spectral curves simulated in this structure was 3.083 × 10^5^–4.671 × 10^19^; therefore, design matrix ***A*** was severely ill conditioned. From Equation 9, a small singular value is the main cause of a larger condition number. To obtain a more stable high-precision reconstruction of the target, first, the singular value sequence is corrected by regularizing the matrix ***G***. Second, using Equation 10, the modified singular value sequence is truncated, smaller singular values are discarded, and 95 % of the singular value sequence is retained.

(Equation 10) $$\frac{\sum_{i=1}^{n}\sigma_{i}^{\prime }}{\sum_{i=1}^{k}\sigma_{i}^{\prime }}\geq 0.95$$

$\sigma_{i}^{\prime }$ is the singular value corrected by the regularization matrix ***G***. Through the earlier calculations, we optimized the condition number correction of 22 sets of absorption spectral curves to 21.73–46.59.

To evaluate the effect of the target spectral reconstruction more comprehensively and with high precision, the squared error (MSE), average absolute error (er), and relative error (E) spectral reconstruction accuracy evaluation criteria were used.

(Equation 11) $$MSE=\frac{1}{n}\sum_{i=1}^{n}{\left(\overset{\wedge }{x_{i}}-x_{i}\right)}^{2},er=\frac{1}{n}\sum_{i=1}^{n}\left|\overset{\wedge }{x_{i}}-x_{i}\right|,E=\frac{{\left\|\overset{\wedge }{x}-x\right\|}_{2}}{{\left\|x\right\|}_{2}}\;\;$$

where $x_{i}$ and x are the original spectral curves, $\overset{\wedge }{x_{i}}$ and $\overset{\wedge }{x}$ are the reconstructed spectral curves, and *n* is the number of spectral bands.

## Discussion

**Figure 9:**
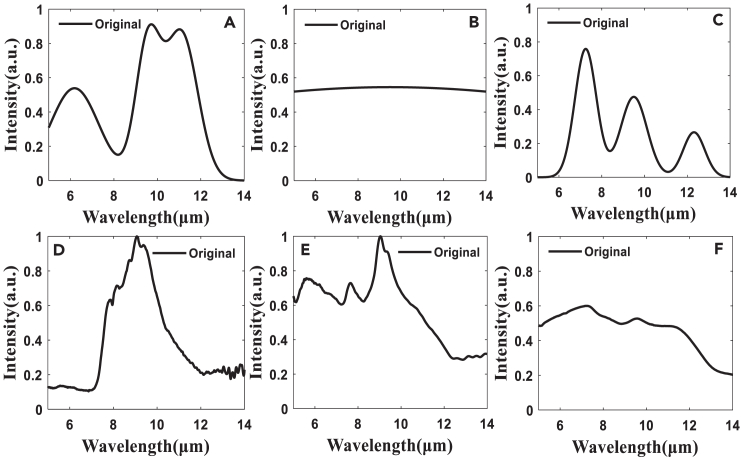
The three simulation targets and three ground targets (A), (B), and (C) show the three simulation targets, and (D), (E), and (F) show the three ground targets.

**Figure 10:**
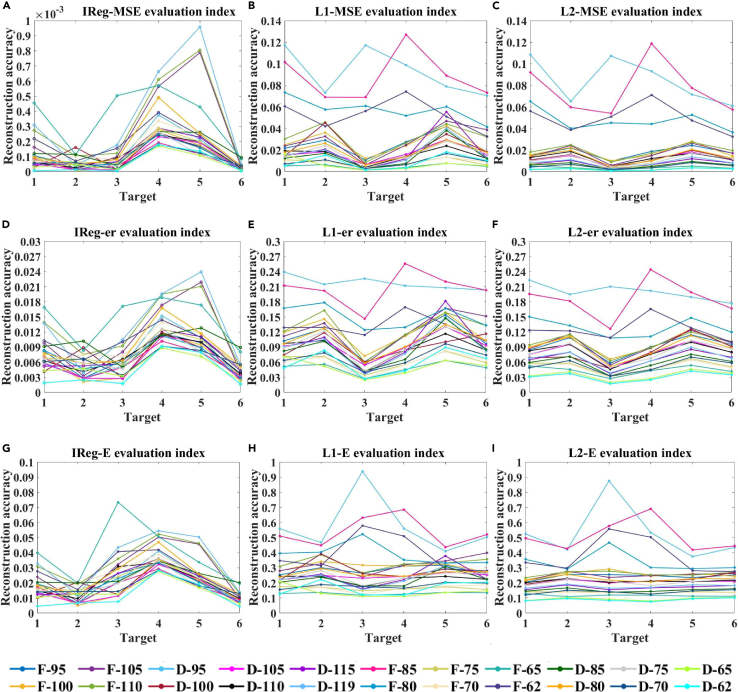
Comparative analysis of spectral curve reconstruction results under 30 dB noise condition MSE, er, and E indices reusing the (A, D, and G) IReg, (B, E, and H) L1, and (C, F, and I) L2 regularization algorithms under a noise level of 30 dB and using a set of 22 different absorption spectral curves as the design matrix.

**Figure 11:**
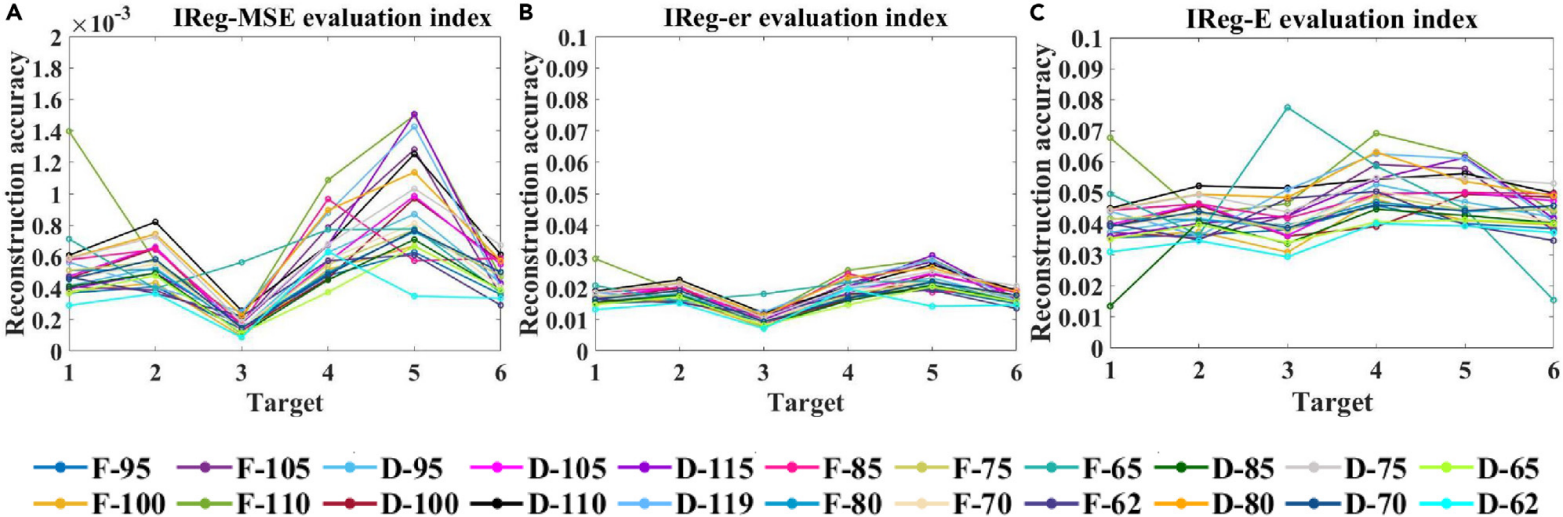
Comparative analysis of spectral curve reconstruction results under 20 dB noise condition MSE, er, and E indices for the (A–C) IReg regularization algorithm under a noise level of 20 dB, using a set of 22 different absorption spectral curves as the design matrix to reconstruct the spectra of three simulation targets and three ground objects.

In this study, the spectra of three simulation targets (A), (B), and (C), as well as three ground objects (D), (E), and (F) shown in Figure 9, were reconstructed using the truncated regularization algorithm based on the singular value ratio of the design matrix and the absorption filter. By examining the effect of data reduction, different numbers of absorption spectral curves were employed as the design matrix ***A***, followed by the application of various reconstruction algorithms. This approach facilitated a precise determination of the design matrix, even after data reduction, leading to high-precision spectral reconstruction. The analysis of the evaluation accuracy of detailed spectral reconstruction is presented in Figures 10 and 11. Detailed data are presented in supplemental information.

Figure 10 depicts the reconstruction accuracy of the IReg, L1, and L2 algorithms for six types of ground objects at an SNR of 30 dB. The MSE, er, and E evaluation indicators of the IReg algorithm are higher than those of the L1 and L2 algorithms (IReg: MSE, er, and E values are less than 9.5829 × 10^−4^, 0.0239, and 0.0734, respectively; L1: MSE, er, and E values are less than 0.1270, 0.2557, and 0.9384, respectively; L2: MSE, er, and E values are less than 0.1187, 0.2438, and 0.8752, respectively). The decrease in the three spectral reconstruction accuracy indicators of the IReg algorithm indicates that this algorithm can improve the accuracy of solving linear ill-conditioned equations, thereby improving the accuracy of spectral reconstruction.

To further evaluate the robustness and accuracy of the IReg algorithm, we conducted spectral reconstruction for six types of ground features at an SNR of 20 dB, and the reconstruction evaluation accuracy is shown in Figure 11. The reconstruction accuracies of the IReg algorithm for MSE, er, and E are less than 1.5073 × 10^−3^, 0.0293, and 0.0775, respectively. A comparison between the reconstruction results obtained at SNRs of 20 and 30 dB reveals that, for the IReg algorithm, the three reconstruction accuracy indicators between the two SNRs exhibit the same values, indicating that the IReg algorithm is highly resistant to noise. Future studies should concentrate on effectively reducing the cost and volume of spectrometers by reducing the number of transmittance curves.

The numbers of curves for D-110°, D-105°, and D-100° are approximately half the number of D-85°, while the number of curves for F-105° is approximately one-third that of D-85°. A comparison between the reconstruction accuracies and results obtained at SNRs of 30 and 20 dB indicates that the IReg algorithm’s reconstruction accuracy and effect are similar at these SNRs. Thus, the IReg algorithm can effectively achieve dimensionality reduction in the number of absorption spectra. In summary, the IReg algorithm improves the accuracy of solving linear ill-conditioned equations by reducing the number of design matrix conditions as well as strengthens the anti-noise ability of the ill-conditioned linear equations. Consequently, this algorithm successfully improves the accuracy of spectral reconstruction. Notably, the IReg algorithm achieves high-precision spectral reconstruction of flat and steep target spectral curves as well as effectively reduces the number of spectral curves and achieves data dimensionality reduction while ensuring the same spectral reconstruction accuracy.

### Conclusion

The absorber designed in this study exhibited several advantages, including polarization sensitivity, insensitivity to structural wear, wide angle coverage, azimuthal ultra-wide-angle performance, high multi-narrow-band absorption, and an adjustable working wavelength. When combined with computational imaging spectroscopy for spectral reconstruction of detected targets, it offers advantages of miniaturization, cost-effectiveness, and robustness against biases. Remarkably, even when reducing the absorption spectrum by up to 1/3, high-precision spectral reconstruction remained achievable for both flat and high-energy steep mid- and long-infrared spectral targets with simultaneous data dimensionality reduction. Therefore, the combination of the infrared multispectral narrow-band absorber and the IReg algorithm effectively reduced the number of absorption spectral curves while maximizing the spectral reconstruction accuracy and thus facilitating a cost-effective analysis. Consequently, this study provides a valuable scientific foundation for future research on the application of miniaturized airborne and spaceborne infrared spectrometers to aerospace resource exploration and infrared target reconnaissance.

### Limitations of the study

In this study, an ultra-wide-angle multispectral narrow-band absorber for infrared spectral reconstruction was designed, and the performance of the micro-nano metamaterial structure absorber was simulated under different wear conditions. In the simulation, we used the tested parameters of the materials in some classical literature for numerical calculation. However, the actual parameters of the materials in the processing and preparation may be different from the parameters in the simulation, and the performance of the absorber may be altered, resulting in reduced absorption efficiency or a shift in the central wavelength. This is the main limitation in the study. Therefore, before the processing and preparation of micro-nano metamaterial absorbers, we first need to test the characteristics of the material. Then the actual parameters of the test are imported into the micro-nano metamaterial structure model for parameter optimization to ensure the reliability of the processed absorber.

## STAR★Methods

### Key resources table

**Table:** 

REAGENT or RESOURCE	SOURCE	IDENTIFIER
**Software and algorithms**		

COMSOL Multiphysics 5.6	COMSOL Co., Ltd.	https://cn.comsol.com/
MATLAB 2019a and 2021a	Mathworks	https://www.mathworks.com/products/matlab.html

**Other**		

ENVI 5.3	ENVI/IDL Resource Center	https://envi.geoscene.cn/

### Resource availability

#### Lead contact

Further information and requests for resources should be directed to and will be fulfilled by the lead contact Yan Zheng (zhengyan19@mails.jlu.edu.cn).

#### Materials availability

For constructing the absorber, we selected the infrared materials ZnS, ZnSe and Ge, which are often used in the infrared spectrum. Because we used the grating compensation method to excite the physical characteristics of surface plasmon resonance, we added a metal material Al to realize the absorber of the grating MDM structure. Therefore, we changed the optical properties of ZnS, ZnSe, Ge, and Al materials by grating the MDM structure and obtained artificial materials with enhanced absorption characteristics, namely metamaterials.

Through analysis, the metamaterial of the grating MDM structure designed in this paper is found to mainly excite one and a variety of plasmon resonance modes through the metal material Al between each layer. However, simultaneously, the Al introduces loss, and weakens the electromagnetic field, which will affect the resonance response of the metamaterial. Therefore, we chose the dielectric material Ge with high refractive index in the infrared material and designed the dielectric position at the top layer to optimize and reduce the non-radiative loss. Because the number of absorption peaks depends on the number of resonance modes in the structure, we chose the infrared materials ZnS and ZnSe with refractive indices between Ge and Al to compose the grating MDM structure and excite the diversity of narrow-band absorption peaks.

#### Data and code availability

• All data reported in this paper will be shared by the lead contact upon reasonable request.
• This paper does not report the original code.
• Any additional information required to reanalyze the data reported in this paper is available from the lead contact upon request.

### Method details

All methods can be found in the main text. Please check the Structural design and simulation section for the design details of the micro–nano structural. Please check the Spectral reconstruction analysis section for the combination of the infrared multispectral narrow-band absorber and IReg algorithm effectively reduced the number of absorption spectral curves while maximizing the spectral reconstruction accuracy. COMSOL Multiphysics is used to design the structure of micro–nano metamaterial absorbers in the manuscript. MATLAB is used to process and analyze spectral reconstruction data in the manuscript.

### Quantification and statistical analysis

The quantification and statistical analysis of the results of the spectral reconstruction can be understood in the Discussion section of the manuscript. Among them, detailed data are presented in Supplemental ITEMS.

## Supplemental information

Document S1. Figures S1‒S8 and Tables S1–S11
