# Supplementary material for: Ultra-wide-angle multispectral narrow-band absorber for infrared spectral reconstruction
Source: iScience. 2024 Apr 8;27(8):109700. doi: 10.1016/j.isci.2024.109700 (PMC11363499; doi:10.1016/j.isci.2024.109700)
Supplement: Document S1. Figures S1‒S8 and Tables S1–S11 [file mmc1.pdf]

iScience, Volume *27*

Supplemental information

Ultra-wide-angle multispectral narrow-band absorber for infrared spectral reconstruction

Yan Zheng, Liu Zhang, Ying Song, Jia-Kun Zhang, and Yong-Nan Lu

Supplemental ITEMS

In the manuscript, Figures. 5(a)–(m) presents the analysed relationship between the narrow-band absorption efficiency and duty ratio for structural gradient angles of β=62–119°; detailed data are presented in Table S1. In the manuscript, Figures. 6(a)–(m) and Table S2 showcase the relationship between narrow-band absorption efficiency and period for structural gradient angles of β=62–119°.

Table S1. [Data analysis of absorption efficiency and duty cycle at structural gradient angles of β=62–119°], **Related to Figure 5.**

Table S1. Data analysis of absorption efficiency and duty cycle at structural gradient angles of β=62–119°

**Gradient angle (°)**	**Multi-peak centre wavelength (μm)**	**Duty cycle**	**Absorption efficiency interval (%)**	**Gradient angle (°)**	**Multi-peak centre wavelength (μm)**	**Duty cycle**	**Absorption efficiency interval (%)**
62°	7-7.7	0.01-0.05	99.98-91.70	65°	6.2-7.8	0.01-0.10	99.72-91.45
	6.6-7.2	0.42-0.48	97.65-90.03		6.9-7.7	0.47-0.54	97.35-90.03
70°	5.9-8.1	0.07-0.19	99.58-90.15	75°	4.3-5	0.05-0.09	98.74-90.18
	4-4.2	0.45-0.57	97.08-82.52		6.1-8.5	0.15-0.28	99.19-90.04
	7.1-8.3	0.51-0.62	98.31-90.10		4.4-5.1	0.49-0.65	99.31-90.57
					7.6-9	0.58-0.70	99.61-90.81
80°	5-9	0.16-0.37	97.82-90.11	85°	4.6-10	0.20-0.49	99.63-90.01
	4.4-5.1	0.59-0.72	99.86-90.08		4.3-5.3	0.64-0.82	99.35-90.01
	8.1-9.8	0.66-0.78	99.60-90.00		8.7-10.9	0.72-0.87	99.46-90.32
95°	4.8-14	0.34-0.85	99.99-91.34	100°	5.2-14	0.43-0.91	99.95-90.33
	7.4-9.8	0.60-0.85	84.84-61.01		7.7-12.5	0.71-0.99	83.62-60.60
105°	5.8-14	0.53-0.99	99.74-92.42	110°	6.9-12.8	0.67-0.99	99.34-94.52
	8.2-11.5	0.77-0.99	73.29-61.73		8-11.1	0.77-0.99	66.78-62.28
115°	4.7-5.8	0.61-0.67	99.01-90.98	119°	4.1-6.9	0.65-0.80	99.41-86.93
	8-11.1	0.81-0.99	99.91-90.01		9-10.2	0.90-0.99	87.00-70.01

Table S2. [Data analysis of absorption efficiency and period at structural gradient angles of β=62–119°], **Related to Figure 6.**

Table S2. Data analysis of absorption efficiency and period at structural gradient angles of β=62–119°

**Gradient angle (°)**	**Multi-peak centre wavelength (μm)**	**Period (μm)**	**Absorption efficiency interval (%)**	**Gradient Angle (°)**	**Multi-peak centre wavelength (μm)**	**Period (μm)**	**Absorption efficiency interval (%)**
62°	7.6-9.6	2.10-3.10	97.68-90.06	65°	7.1-10.2	1.85-3.40	99.64-90.21
	7.6-10.5	2.10-3.50	97.68-80.68		7-11.1	1.75-3.80	99.64-80.01
	6.9-10	3.15-5.30	98.73-90.07		6-10.1	2.70-5.55	99.88-90.05
	6.4-11.2	2.80-6.10	98.73-80.01		6-11.2	2.65-6.30	99.88-80.0
70°	7.4-10.7	2.20-3.70	99.98-90.03	75°	8.6-9.8	1.05-1.40	94.39-90.02
	6.3-11.9	1.40-4.20	99.98-80.01		8.6-10.2	1.05-1.50	94.39-82.38
	9.2-10.9	5.25-6.40	83.58-80.04		8-11.5	2.60-4.10	99.42-90.59
					7.3-12.3	2.25-4.45	99.42-80.08
80°	7.5-9.9	1.10-1.70	99.84-90.04	85°	7.5-10.1	1.40-2.00	99.94-90.07
	7-10.4	1.00-1.80	99.84-80.54		6.9-10.5	1.26-2.10	99.94-80.13
	8.5-12.3	2.90-4.50	99.98-90.86		7.5-13	3.15-4.85	99.78-90.01
	7.7-13.1	2.55-4.85	99.98-81.06		6.9-13.9	2.80-5.25	99.78-80.34
95°	9.8-14	3.70-5.50	99.73-90.02	100°	10.3-14	4.00-5.55	99.90-90.18
	8.8-14	3.30-5.45	99.73-80.76		9.2-14	3.55-5.55	99.90-80.92
	7.1-10.5	1.85-2.60	99.54-90.67		5.3-10.7	1.75-2.90	99.95-90.95
	4.7-10.9	1.35-2.70	99.54-82.65		5.1-11.3	1.70-3.05	99.95-82.12
105°	10.8-14	4.30-5.60	99.94-90.30	110°	11.4-14	4.65-5.75	99.83-90.84
	9.6-14	3.80-5.60	99.94-80.08		9.9-14	4.05-5.80	99.83-80.18
	5.7-11.2	2.10-3.30	99.83-90.31		6.6-11.7	2.60-3.70	99.93-90.05
	5.7-11.7	2.10-3.40	99.83-82.03		6.4-12.3	2.55-3.85	99.93-80.94
115°	11.8-14	4.95-5.95	99.45-90.78	119°	12-14	5.15-6.00	97.78-90.16
	10.3-14	4.35-6.00	99.45-80.93		10.5-14	4.55-6.05	97.78-80.71
	7.7-12	3.15-4.10	99.41-91.67		8.7-12.8	3.65-4.55	97.92-90.01
	7.5-12.9	3.10-4.30	99.41-82.18		8.4-13.7	3.60-4.75	97.92-80.34

Table S3. [Wavelength range and number of spectral curves for different structural gradient angles], **Related to Figures 5 and 6.**

Table S3. Wavelength range and number of spectral curves for different structural gradient angles

**Structural gradient under the duty cycle condition**	**wavelength range (μm)**	**Number of spectral curves**	**Structural gradient under the periodic condition**	**wavelength range (μm)**	**Number of spectral curves**
F-110°	6.5-13	32	D-119°	8-14	55
F-105°	6-14	47	D-115°	7-14	61
F-100°	5-14	58	D-110°	6-14	70
F-95°	5-14	65	D-105°	6-14	72
F-85°	5-14	69	D-100°	6-14	74
F-80°	5-12	67	D-95°	6-14	79
F-75°	4.7-10.5	66	D-85°	6-14	151
F-70°	4.7-9.5	58	D-80°	6-14	89
F-65°	6-9	39	D-75°	6-14	91
F-62°	6-9	32	D-70°	6.3-13.5	113
			D-65°	6.5-12.5	105
			D-62°	7-12.5	100

This paper presents the reconstruction results of four sets of transmittance spectral curves, i.e. D-85°, D-100°, F-105°, and F-100°, for six target spectral curves at SNRs of 30 and 20 dB, as shown in Figures. 1-8 and Tables S4-S11. These results indicate that at SNR = 30 dB, significant jitter is observed in the reconstruction curves obtained using the L1 and L2 algorithms; specifically, for the smooth target spectral curves, the reconstruction accuracy is poor. However, the reconstruction performance and accuracy of the IReg algorithm is significantly higher than those of the L1 and L2 algorithms. At an SNR of 20 dB, the IReg algorithm can still reconstruct the six target spectral curves with a high precision, which reflects the anti-noise characteristic of this algorithm.

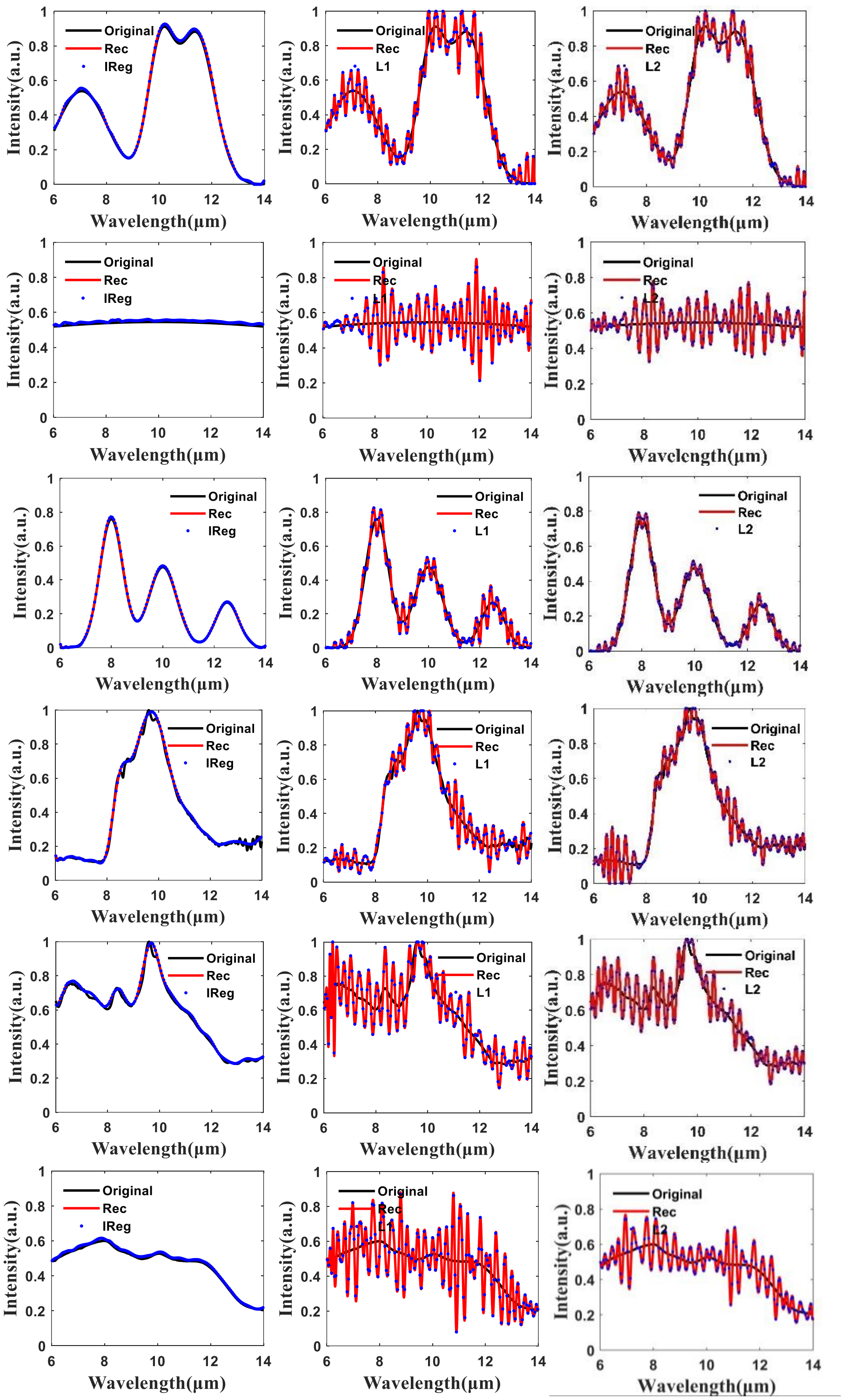

Figure S1. Comparative analysis of spectral reconstruction results

D-85° absorption-type spectral curve data analysis of the accuracy MSE, er, and E of spectral reconstruction of three simulation targets and three ground targets under the wavelength of 6-14 μm interval and 30 dB noise, **Related to Figure 10.**

Table S4. [Absorption spectral curves of the D-85° group and spectral reconstruction data analysis under a 6-14 μm interval and 30 dB noise], **Related to Figure 10.**

Table S4. Absorption spectral curves of the D-85° group and spectral reconstruction data analysis under a 6-14 μm interval and 30 dB noise

**D-85°/ 30dB**		**Simulation target**			**Ground object target**		
**Evaluation of accuracy of spectral reconstruction**		**MSE mean**	**er mean**	**E mean**	**MSE mean**	**er mean**	**E mean**
target 1	IReg	1.1949×10^{-4}	9.1265×10^{-3}	0.0205	2.6459×10^{-4}	0.0114	0.0345
	L1	0.0125	0.0821	0.2044	7.5293×10^{-3}	0.0654	0.1790
	L2	6.0872×10^{-3}	0.0593	0.1451	4.6847×10^{-3}	0.0536	0.1430
target 2	IReg	1.1381×10^{-4}	0.0102	0.0199	2.6042×10^{-4}	0.0128	0.0262
	L1	0.0170	0.1014	0.2379	0.0383	0.1471	0.3083
	L2	8.2512×10^{-3}	0.0714	0.1670	9.4216×10^{-3}	0.0755	0.1555
target 3	IReg	4.0893×10^{-5}	4.9745×10^{-3}	0.0209	9.3396×10^{-5}	8.9141×10^{-3}	0.0202
	L1	3.1586×10^{-3}	0.0425	0.1809	0.0119	0.0853	0.2236
	L2	1.8276×10^{-3}	0.0325	0.1381	6.1004×10^{-3}	0.0613	0.1608

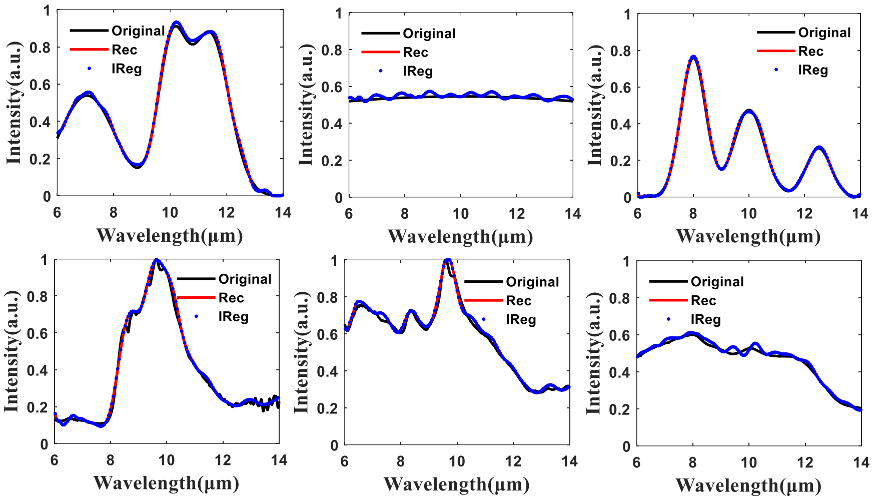

Figure S2. Comparative analysis of spectral reconstruction results

D-85° data analysis of the spectral reconstruction evaluation accuracy MSE, er, E and three ground targets in the 6-14 μm wavelength interval and at 20 dB noise, **Related to Figure 11.**

Table S5. [Spectral curves of the D-85° group and spectral reconstruction data analysis under the 6-14 μm interval and 20 dB noise], **Related to Figure 11.**

Table S5. Spectral curves of the D-85° group and spectral reconstruction data analysis under the 6-14 μm interval and 20 dB noise

D-85°/ 20dB	Simulation target			Ground object target		
Evaluation of accuracy of spectral reconstruction	MSE Mean	er Mean	E mean	MSE Mean	er Mean	E mean
target 1- IReg	4.0958×10^{-4}	0.0155	0.0135	4.5203×10^{-4}	0.0160	0.0448
target 2- IReg	4.9519×10^{-4}	0.0177	0.0407	7.0872×10^{-4}	0.0201	0.0427
target 3- IReg	1.0876×10^{-4}	8.0496×10^{-3}	0.0337	3.8534×10^{-4}	0.0157	0.0403

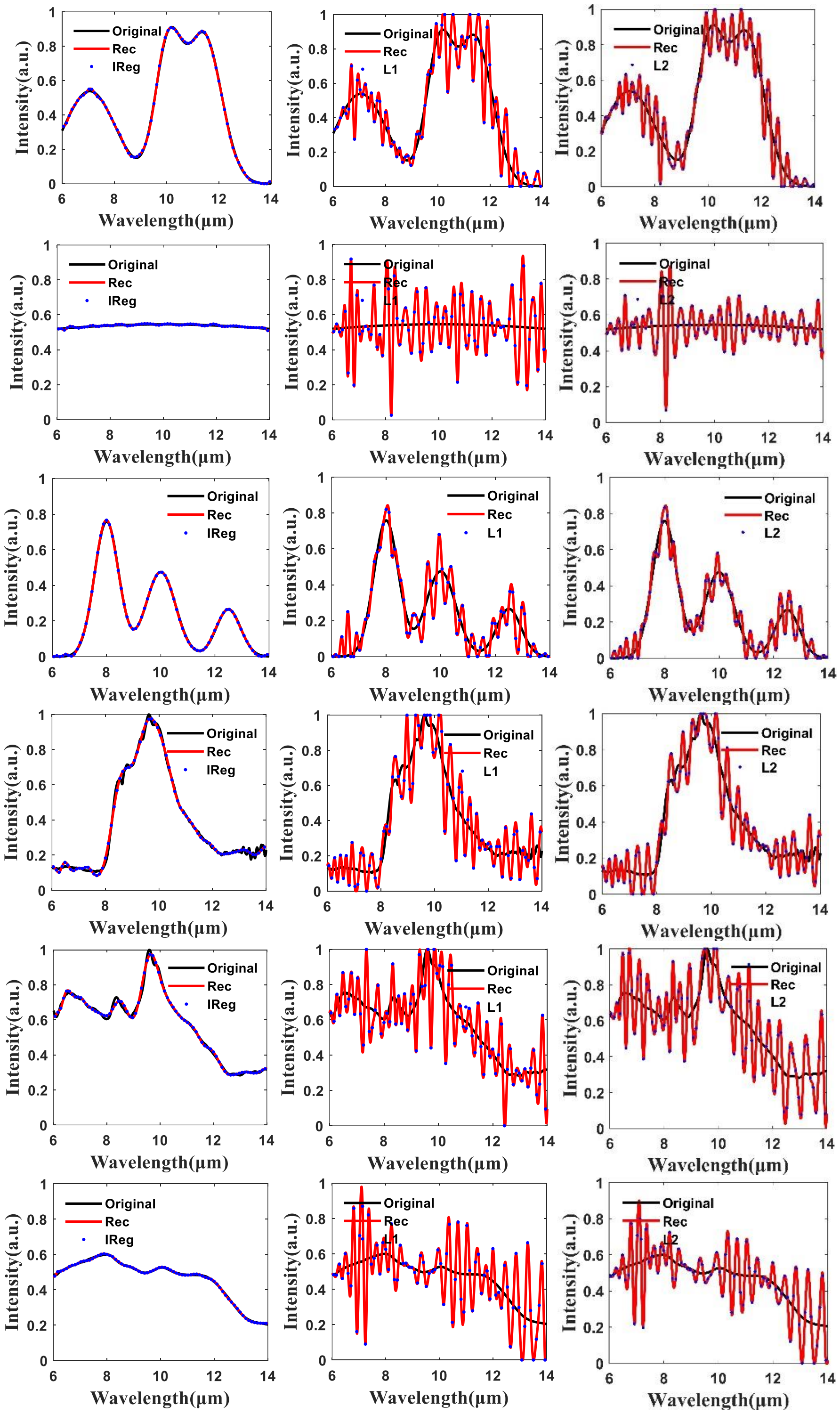

Figure S3. Comparative analysis of spectral reconstruction results

D-100° absorption-type spectral curve data analysis of the accuracy MSE, er, and E of spectral reconstruction of three simulation targets and three ground targets under the wavelength of 6-14 μm interval and 30 dB noise, **Related to Figure 10.**

Table S6. [Absorption spectral curves of the D-100° group and spectral reconstruction data analysis under a 6-14 μm interval and 30 dB noise], **Related to Figure 10.**

Table S6. Absorption spectral curves of the D-100° group and spectral reconstruction data analysis under a 6-14 μm interval and 30 dB noise

D-100°/ 30dB		Simulation target			Ground object target		
Evaluation of accuracy of spectral reconstruction		MSE mean	er mean	E mean	MSE mean	er mean	E mean
target 1	IReg	2.8891×10^{-5}	4.1386×10^{-3}	0.0101	2.5883×10^{-4}	0.0119	0.0341
	L1	0.0145	0.0892	0.2183	0.0140	0.0916	0.2422
	L2	0.0108	0.0785	0.1904	0.0103	0.0799	0.2113
target 2	IReg	1.1378×10^{-5}	2.6238×10^{-3}	6.2098×10^{-3}	1.6013×10^{-4}	8.8777×10^{-3}	0.0206
	L1	0.0452	0.1674	0.3922	0.0348	0.1446	0.2918
	L2	0.0159	0.0983	0.2289	0.0198	0.1103	0.2243
target 3	IReg	1.4211×10^{-5}	2.7940×10^{-3}	0.0123	1.1145×10^{-5}	2.6241×10^{-3}	6.9064×10^{-3}
	L1	6.5225×10^{-3}	0.0620	0.2615	0.0181	0.1025	0.2693
	L2	3.8490×10^{-3}	0.0469	0.1988	0.0120	0.0854	0.2233

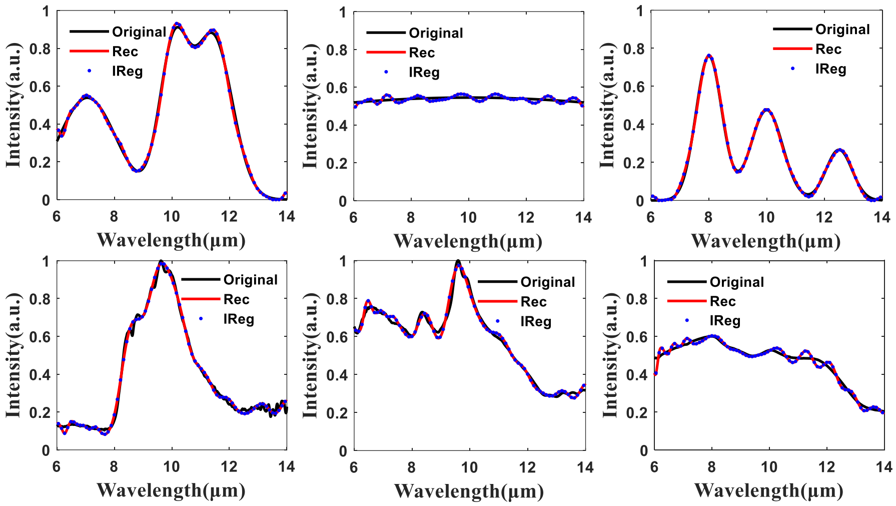

Figure S4. Comparative analysis of spectral reconstruction results

D-100° data analysis of the spectral reconstruction evaluation accuracy MSE, er, E and three ground targets in the 6-14 μm wavelength interval and at 20 dB noise, **Related to Figure 11.**

Table S7. [Absorption spectral curves of the D-100° group and spectral reconstruction data analysis under a 6-14 μm interval and 20 dB noise], **Related to Figure 11.**

Table S7. Absorption spectral curves of the D-100° group and spectral reconstruction data analysis under a 6-14 μm interval and 20 dB noise

D-100°/ 20dB	Simulation target			Ground object target		
Evaluation of accuracy of spectral reconstruction	MSE Mean	er Mean	E mean	MSE Mean	er Mean	E mean
target 1- IReg	4.6139×10^{-4}	0.0164	0.0391	4.6139×10^{-4}	0.0164	0.0391
target 2- IReg	6.5132×10^{-4}	0.0200	0.0460	6.5132×10^{-4}	0.0244	0.0496
target 3- IReg	1.2798×10^{-4}	8.8561×10^{-3}	0.0361	5.7013×10^{-4}	0.0188	0.0487

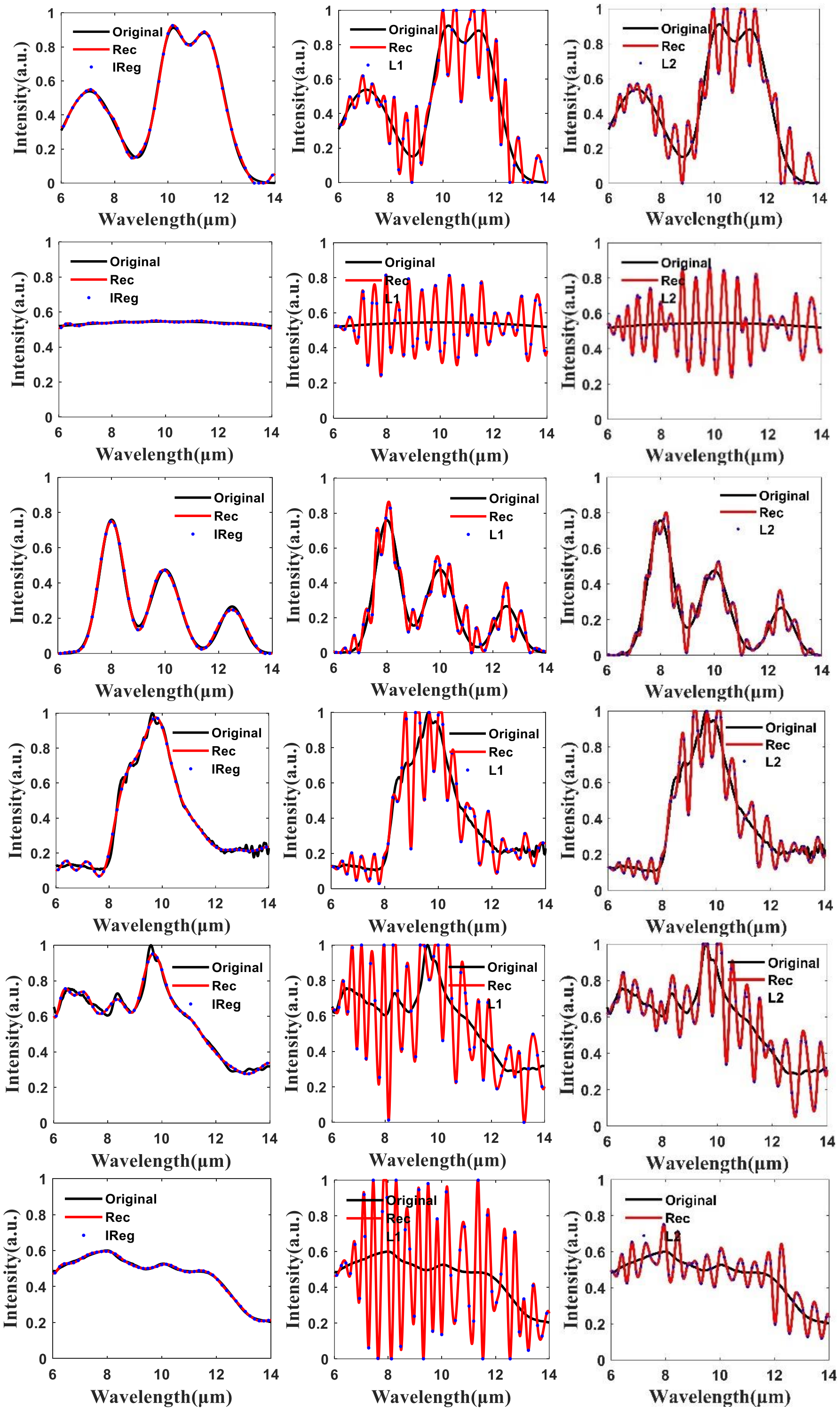

Figure S5. Comparative analysis of spectral reconstruction results

F-105°absorption-type spectral curve data analysis of the accuracy MSE, er, and E of spectral reconstruction of three simulation targets and three ground targets under the wavelength of 6-14 μm interval and 30 dB noise, **Related to Figure 10.**

Table S8. [Absorption spectral curves of the F-105° group and spectral reconstruction data analysis under a 6-14 μm interval and 30 dB noise], **Related to Figure 10.**

Table S8. Absorption spectral curves of the F-105° group and spectral reconstruction data analysis under a 6-14 μm interval and 30 dB noise

F-105°/ 30dB		Simulation target			Ground object target		
Evaluation of accuracy of spectral reconstruction		MSE mean	er mean	E mean	MSE mean	er mean	E mean
target 1	IReg	1.6143×10^{-4}	9.7851×10^{-3}	0.0238	5.6428×10^{-4}	0.0173	0.0503
	L1	0.0238	0.1114	0.2803	0.0248	0.1137	0.3189
	L2	0.0137	0.0862	0.2131	0.0149	0.0884	0.2471
target 2	IReg	1.7611×10^{-5}	3.2496×10^{-3}	7.7996×10^{-3}	7.8832×10^{-4}	0.0219	0.0456
	L1	0.03262	0.1375	0.3265	0.0469	0.1670	0.3452
	L2	0.02423	0.1158	0.2773	0.0263	0.1232	0.2575
target 3	IReg	9.7994×10^{-5}	8.0324×10^{-3}	0.0324	2.3250×10^{-5}	4.0151×10^{-3}	0.0101
	L1	6.1516×10^{-3}	0.0553	0.2392	0.0388	0.1513	0.3989
	L2	5.7551×10^{-3}	0.0544	0.2363	0.0170	0.0986	0.2617

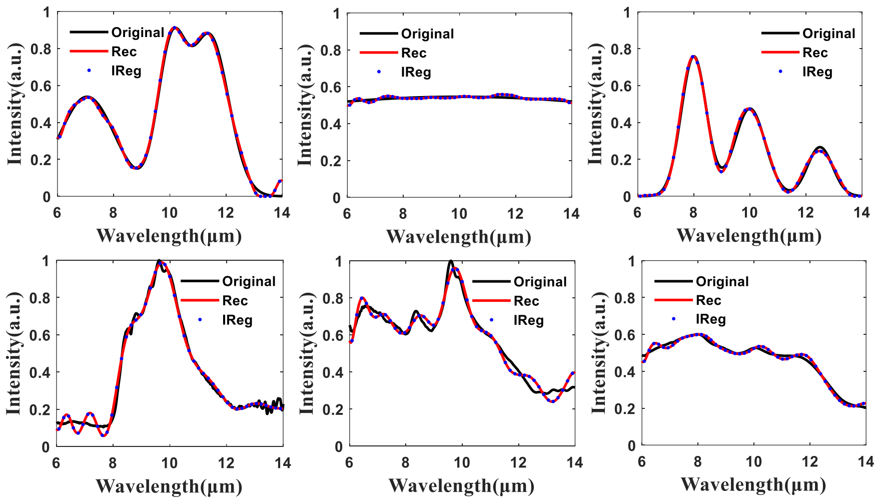

Figure S6. Comparative analysis of spectral reconstruction results

F-105° data analysis of the spectral reconstruction evaluation accuracy MSE, er, E and three ground targets in the 6-14 μm wavelength interval and at 20 dB noise, **Related to Figure 11.**

Table S9. [Absorption spectral curves of the F-105° group and spectral reconstruction data analysis under a 6-14 μm interval and 20 dB noise], **Related to Figure 11.**

Table S9. Absorption spectral curves of the F-105° group and spectral reconstruction data analysis under a 6-14 μm interval and 20 dB noise

F-105°/ 20dB	Simulation target			Ground object target		
Evaluation of accuracy of spectral reconstruction	MSE Mean	er Mean	E mean	MSE Mean	er Mean	E mean
target 1- IReg	4.0808×10^{-4}	0.0159	0.0373	7.8655×10^{-4}	0.0215	0.0592
target 2- IReg	3.8691×10^{-4}	0.0155	0.0354	1.2812×10^{-4}	0.0284	0.0578
target 3- IReg	1.7258×10^{-4}	0.0104	0.0427	3.5459×10^{-4}	0.0148	0.0379

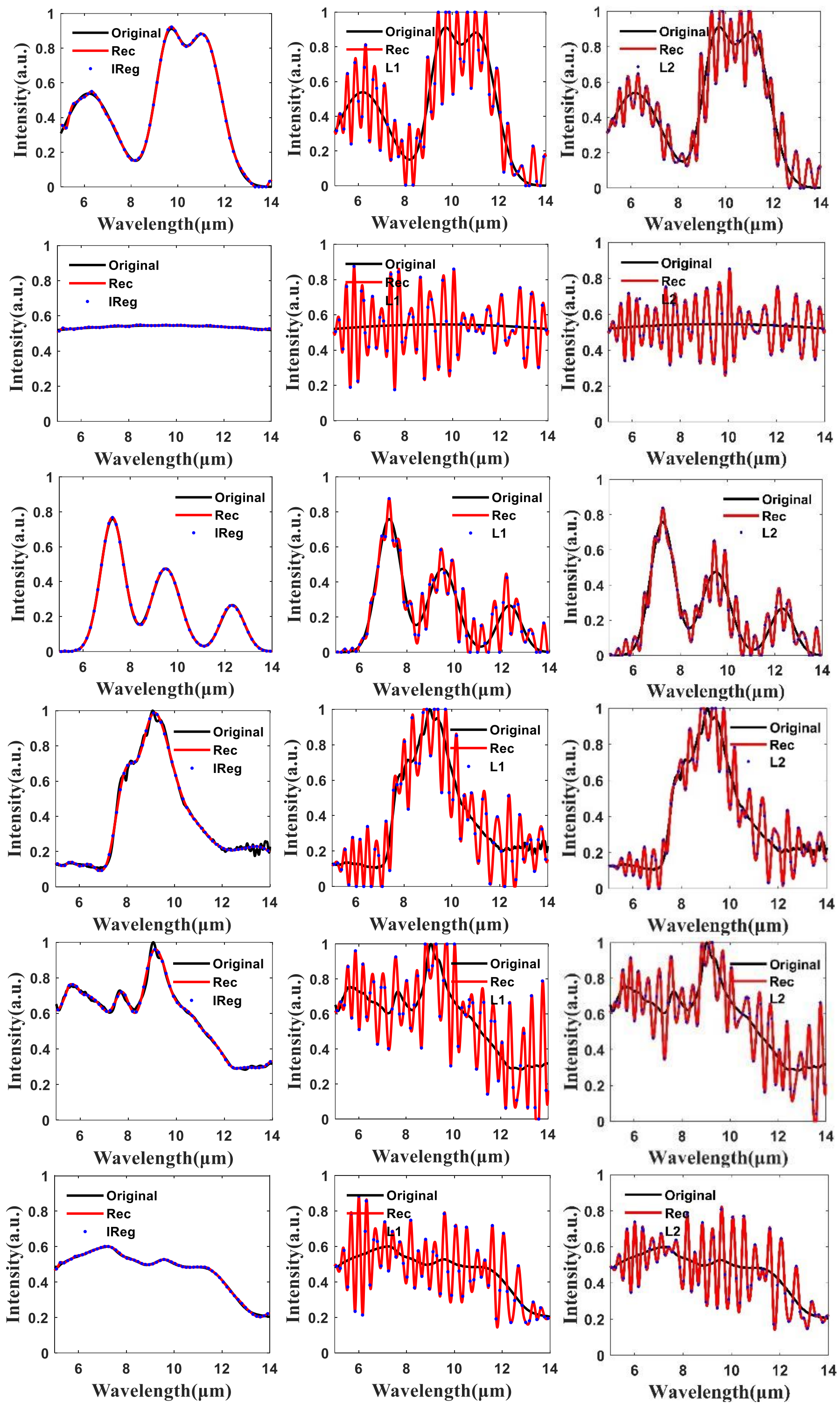

Figure S7. Comparative analysis of spectral reconstruction results

F-100° absorption-type spectral curve data analysis of the accuracy MSE, er, and E of spectral reconstruction of three simulation targets and three ground targets under the wavelength of 5-14 μm interval and 30 dB noise, **Related to Figure 10.**

Table S10. [Absorption spectral curves of the F-100° group and spectral reconstruction data analysis under a 5-14 μm interval and 30 dB noise], **Related to Figure 10.**

Table S10. Absorption spectral curves of the F-100° group and spectral reconstruction data analysis under a 5-14 μm interval and 30 dB noise

F-100°/ 30dB		Simulation target			Ground object target		
Evaluation of accuracy of spectral reconstruction		MSE mean	er mean	E mean	MSE mean	er mean	E mean
target 1	IReg	9.2587×10^{-5}	7.2328×10^{-3}	0.0181	2.8065×10^{-4}	0.0117	0.0355
	L1	0.0249	0.1193	0.2781	0.0240	0.1117	0.3123
	L2	0.0158	0.0898	0.2196	0.0153	0.0873	0.2440
target 2	IReg	7.4921×10^{-6}	2.1399×10^{-3}	5.0266×10^{-3}	1.7248×10^{-4}	9.3927×10^{-4}	0.0214
	L1	0.0362	0.1455	0.3413	0.0423	0.1581	0.3238
	L2	0.0224	0.1125	0.2665	0.0282	0.1260	0.2605
target 3	IReg	1.2399×10^{-5}	2.8016×10^{-3}	0.0115	1.9653×10^{-5}	3.2927×10^{-3}	9.2394×10^{-3}
	L1	0.0122	0.0721	0.3172	0.0178	0.1020	0.2701
	L2	0.0101	0.0662	0.2914	0.0144	0.0918	0.2439

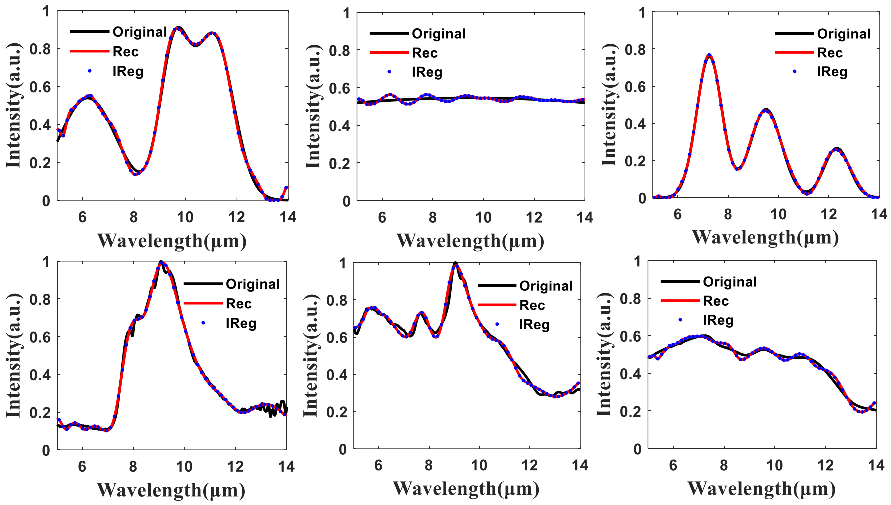

Figure S8. Comparative analysis of spectral reconstruction results

F-100° data analysis of the spectral reconstruction evaluation accuracy MSE, er, E and three ground targets in the 5-14 μm wavelength interval and at 20 dB noise, **Related to Figure 11.**

Table S11. [Absorption spectral curves of the F-100° group and spectral reconstruction data analysis under a 5-14 μm interval and 20 dB noise], **Related to Figure 11.**

Table S11. Absorption spectral curves of the F-100° group and spectral reconstruction data analysis under a 5-14 μm interval and 20 dB noise

F-100°/ 20dB	Simulation target			Ground object target		
Evaluation of accuracy of spectral reconstruction	MSE Mean	er Mean	E mean	MSE Mean	er Mean	E mean
target 1- IReg	3.7865×10^{-4}	0.0151	0.0358	5.1858×10^{-4}	0.0176	0.0479
target 2- IReg	4.3234×10^{-4}	0.0165	0.0374	6.6758×10^{-4}	0.0202	0.0411
target 3- IReg	9.3078×10^{-5}	7.5082×10^{-3}	0.0309	3.8552×10^{-4}	0.0156	0.0398
